# Strategic Negotiation Factors Influencing Recreational Sport Participation and Urban Wellbeing

**DOI:** 10.3390/healthcare14111553

**Published:** 2026-06-02

**Authors:** Georgia Yfantidou, Alexia Noutsou, Eleni Spyridopoulou, Panagiota Balaska

**Affiliations:** 1 Department of Physical Education and Sport Science, Democritus University of Thrace, 69100 Komotini, Greece; anoutsou@gmail.com (A.N.); espyrido@phyed.duth.gr (E.S.); 2 Department of Physical Education and Sport Science, Aristotle University of Thessaloniki, 57101 Thessaloniki, Greece; pmpalask@phed.auth.gr

**Keywords:** negotiation strategies, recreation participation, recreational sports, well-being, urban green spaces, green exercise, PERMA profiler, urban recreation

## Abstract

Background: Physical activity in urban green environments contributes to both physical and psychological well-being. Although negotiation strategies help individuals overcome barriers to participation in recreational sport, their interaction with environmental factors such as urban green spaces remains underexplored. This study examines the relationship between negotiation strategies and well-being among urban residents and introduces “Green Commitment” to capture engagement with green exercise environments. Methods: The sample consisted of 233 adults (28.8% men, 71.2% women) aged 19–77 years living in Athens. Data was collected using the Negotiation Strategies Scale (33 items across eleven dimensions) and an adapted PERMA Profiler, which assesses well-being across five dimensions: positive emotions, engagement, relationships, meaning, and achievement. Six additional items measured engagement with urban green environments. Exploratory factor analysis, reliability analysis, descriptive statistics, ANOVA, MANOVA, and Pearson correlations were conducted using SPSS v.29. Results: The analysis confirmed satisfactory reliability and a five-factor structure of well-being, including Green Commitment, explaining 64.97% of total variance. Self-motivation recorded the highest mean value (M = 6.2). Significant positive correlations were found between most negotiation strategy dimensions and well-being, particularly for physical health and engagement–achievement (e.g., r = 0.469). Demographic differences were also observed. Conclusions: Negotiation strategies facilitate participation in recreational sport and enhance well-being in urban populations. Engagement with urban green environments, reflected in Green Commitment, further supports these outcomes. The study offers an integrated framework linking behavioral strategies, environmental context, and well-being.

## 1. Introduction

Regular participation in physical activity and recreational sport is widely recognized as an important contributor to both physical health and psychological well-being. Beyond individual health benefits, engagement in recreational activities may also strengthen social interaction, improve quality of life, and support healthier urban communities [1]. Physical activity refers to any bodily movement produced by skeletal muscles that results in energy expenditure. Exercise is a subcategory of physical activity that is planned, structured, and repetitive, with the objective of improving or maintaining physical fitness. In contrast, sport typically involves organized, competitive, and rule-governed activities that may require specific skills and often include social or institutional frameworks. These distinctions are important for understanding different forms of participation and their relationship with well-being. From a physical health perspective, regular participation in physical activity contributes to improvements in functional capacity and overall physical condition over time [2]. At the level of well-being, participation in recreational activities provides enjoyment and satisfaction derived from hedonic experiences [3,4,5], while psychologically it enhances self-esteem and strengthens individuals’ sense of goal achievement [6]. Furthermore, regular physical activity plays an important role in the prevention of chronic diseases such as obesity, cancer, diabetes, and cardiovascular diseases [7,8].

Participation in recreational sport activities also has a strong social dimension [9]. Individuals often organize themselves into groups with the aim of sharing experiences and developing meaningful social interactions [10]. In response to the importance of physical activity for public health, several countries have developed policies to improve access to both indoor and outdoor sport facilities for all social and age groups in order to enhance citizens’ quality of life [11]. Given the growing recognition of physical activity as a means of promoting both individual and social well-being, research interest has increasingly focused on understanding the internal mechanisms that encourage participation in recreational sport activities with the ultimate aim of improving people’s well-being [12,13].

One of the most important internal factors influencing the development of positive attitudes and behavioral commitment toward exercise is negotiation strategies [14]. Several studies in the field of sport and recreation have emphasized the concept of negotiation strategies [15,16], considering them complementary to the behavioral change process described in the Transtheoretical Model [17]. Behavioral changes occur at both cognitive and behavioral levels [18] and lead to the development of positive cognitive and emotional attitudes [19]. These attitudes enable individuals to manage negotiation strategies more effectively and promote sustainable participation in recreational sport activities [20].

Positive cognitive and emotional attitudes refer to individuals’ perceptions of the beneficial effects of exercise and to the emotions experienced through participation [17]. Urban green spaces that provide opportunities for recreation may function as important environmental factors that encourage positive emotional attitudes toward physical activity. Participation in outdoor recreational activities has been associated with positive experiences [21] and the development of emotions that promote personal well-being [10]. At the same time, modern lifestyles and increasing disconnection from the natural environment highlight the need to examine the impact of urban green recreational spaces on citizens’ lives [22].

The integration of the Constraint Negotiation Theory with the Transtheoretical Model in the study of strategies individuals use to overcome barriers and change behavior led to the development of a more detailed measurement scale consisting of 33 items structured across 11 dimensions [12], as follows: Knowledge, Negative Impact Understanding by Non-Exercise, Self-motivation, Enable, Socialization, Enhancement, Commitment, Create Pulse, Develop Relations for Encouragement, Time, and Financial. Each dimension reflects different cognitive and behavioral processes individuals employ to facilitate participation in physical activity.

In the context of the present study, recreational sport participation is considered a structured form of physical activity, whereas exercise may occur independently of sport and may or may not take place within urban green environments. Green spaces provide opportunities for both structured (sport) and unstructured (exercise or general physical activity) engagement, and their role in promoting well-being is examined across these contexts. Individuals apply negotiation strategies to increase or maintain their participation in exercise. Through these strategies, they expand their knowledge about the benefits of physical activity and recognize the positive emotions associated with participation [23]. For example, individuals may seek information from books and magazines related to exercise, reflect on the health consequences of inactivity, encourage themselves to exercise, and use physical activity as a way to overcome daily inactivity. They may also organize their schedules to make time for exercise, manage financial costs related to participation, place sports equipment in visible locations as reminders, and reward themselves for engaging in physical activity [22]. Participation in sport may also be influenced by financial factors such as membership fees, equipment costs, and transportation, which can act as barriers to engagement. In addition, both intrinsic motivation (e.g., enjoyment, personal satisfaction) and extrinsic motivation (e.g., social recognition, health benefits) play a significant role in shaping individuals’ decisions to participate in sport and physical activity.

Building on these behavioral processes, the concept of well-being provides a broader framework for understanding how participation in recreational sport activities influences individuals’ overall quality of life. The concept of well-being is often used interchangeably with health [24] and encompasses interconnected dimensions including physical, emotional, spiritual, social, and environmental aspects that continuously interact with one another. According to [25], well-being can be defined as a “healthy lifestyle” that includes regular physical activity, proper nutrition, elimination of unhealthy behaviors, and the maintenance of positive emotional health. 

Ref. [26] suggested that subjective well-being (SWB) reflects the balance between positive and negative emotional experiences and includes three main components: frequent positive emotions, relatively low levels of negative emotions, and overall life satisfaction [27]. Another influential theory of well-being is the PERMA model [28], which includes five domains: positive emotions, engagement, relationships, meaning, and achievement. These components collectively capture the multidimensional nature of human well-being.

The PERMA model combines both hedonic and eudaimonic aspects and provides a comprehensive framework for understanding well-being [29]. Research has shown that participation in physical activity can positively influence well-being by promoting positive emotions, enhancing self-esteem, and increasing individuals’ sense of competence and achievement [30]. In this context, the physical and environmental settings in which activity takes place—particularly urban green spaces—play a crucial role in shaping well-being outcomes.

Urban green environments offer unique opportunities for engaging in both structured and unstructured forms of physical activity, thereby enhancing the multidimensional aspects of well-being. Urban green spaces provide opportunities for both structured and unstructured physical activity and have been associated with improved psychological well-being, stress reduction, and healthier lifestyles [31,32,33].

The development of green recreational areas supports an active lifestyle and encourages sustainable engagement with physical activity by reconnecting individuals with the natural environment [34,35]. Studies have linked access to open recreational spaces with improved sustainability and quality of life [36]. Psychologically, urban green spaces provide opportunities for relaxation, stress reduction, and the experience of positive emotions [37]. For example, a Swedish study demonstrated that increased time spent in outdoor recreational environments was associated with reduced stress levels [38].

In order to understand well-being, both subjective indicators (e.g., happiness and emotional states) and objective indicators (e.g., housing conditions, education level, and life expectancy) must be considered [39]. Given the importance of physical activity for well-being, the present study incorporated six additional questions into the PERMA questionnaire in order to assess the impact of outdoor green exercise spaces on citizens’ well-being.

In this context, the present study contributes to the literature by integrating negotiation strategies theory with the PERMA model of well-being within the framework of urban green recreation. While previous research has examined physical activity, well-being, and urban green spaces separately, limited attention has been given to how behavioral strategies used to overcome participation barriers influence well-being outcomes in urban environments. By examining negotiation strategies alongside well-being dimensions, this study provides insight into the behavioral mechanisms through which individuals sustain participation in recreational sport activities. Furthermore, the introduction of the dimension “Green Commitment” expands the conceptualization of well-being by incorporating individuals’ engagement with urban green environments as an additional component of flourishing. The concept of “Green Commitment” refers to the extent to which individuals actively engage with and value participation in physical activity within urban green environments. High levels of green commitment are reflected in regular use of parks and outdoor spaces, preference for exercising in natural settings, and awareness of the psychological and physical benefits of such environments. In contrast, low levels of green commitment may involve limited interaction with green spaces or preference for indoor or non-nature-based activities. Factors influencing green commitment may include accessibility, environmental awareness, and personal motivation. Therefore, the study offers a more comprehensive framework for understanding the interaction between behavioral strategies, environmental contexts, and well-being in contemporary urban societies.

Despite the growing body of research on physical activity, well-being, and urban green environments, these domains are often examined in isolation. In particular, limited attention has been given to the behavioral mechanisms through which individuals overcome participation barriers in urban contexts and how these mechanisms interact with environmental factors to influence well-being outcomes. To address this gap, the present study integrates negotiation strategies theory with the PERMA model of well-being within the context of urban green recreation. Furthermore, this study introduces the concept of “Green Commitment” as an additional dimension reflecting individuals’ engagement with and psychological connection to urban green exercise environments. Rather than redefining the PERMA model, Green Commitment is conceptualized as a context-specific extension that captures the role of environmental engagement in shaping well-being in urban settings.

### 1.1. Research Aim

**Figure 1:**
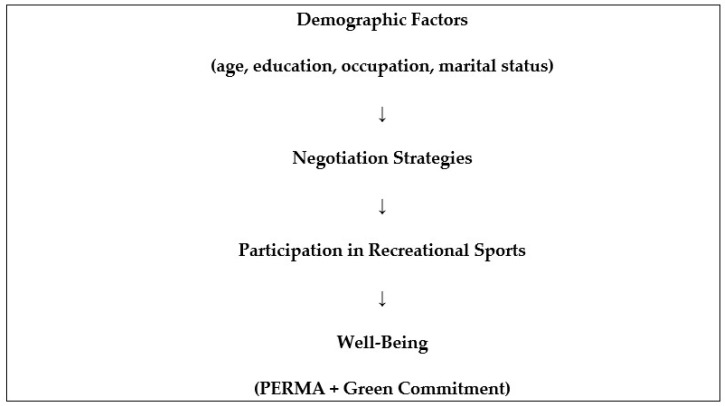
Conceptual framework illustrates the relationships between demographic factors, negotiation strategies, recreational sport participation, and well-being in urban green environments.

The aim of this study was to examine the role of negotiation strategies in facilitating participation in recreational sport activities and their relationship with well-being among urban residents. In addition, the study explores how engagement with urban green environments supports both structured (sport) and unstructured (exercise and general physical activity) forms of participation, and how these interactions contribute to well-being outcomes (Figure 1).

### 1.2. Research Hypotheses

**H1.**  *There are differences in the evaluation of well-being according to the demographic characteristics of the sample.*

**H2.**  *There are differences in the evaluation of negotiation strategies according to the demographic characteristics of the sample.*

**H3.**  *There is a correlation between the dimensions of negotiation strategies and the dimensions of well-being.*

## 2. Materials and Methods

### 2.1. Participants

The sample consisted of 233 adults residing in the urban area of Athens, including 67 men and 166 women.

### 2.2. Measurement Instruments

A quantitative research design was applied, and two questionnaires were distributed. (A) To evaluate negotiation strategies, the questionnaire developed by [12] was used. The instrument consists of 33 items structured across eleven dimensions: Knowledge, Negative Impact Understanding by Non-Exercise, Self-motivation, Enable, Socialization, Enhancement, Commitment, Create Pulse, Develop Relations for Encouragement, Time, and Financial, with each dimension comprising three items. The structural validity and reliability of the questionnaire scales have been confirmed in previous studies with satisfactory psychometric indicators [17]. (B) To assess well-being, the PERMA Profiler questionnaire [40] was used. The instrument was translated and validated for the Greek population by [41]. The original questionnaire consists of 23 items; however, for the purposes of the present study, six additional items related to green environments were added. The inclusion of these six additional items related to green exercise environments was intended to explore the role of environmental engagement in well-being within an urban context. These items were treated as exploratory and subjected to factor analysis alongside the original PERMA items. The emergence of the “Green Commitment” factor should therefore be interpreted as a context-specific extension rather than a validated modification of the original PERMA structure. Further research is required to establish the validity and reliability of this dimension in different populations. Consequently, the final questionnaire consisted of 29 items structured into five dimensions: Green Commitment, Engagement–Achievement, Positive Emotions–Happiness, Negative Emotions–Loneliness, and Physical Health. Responses to both questionnaires were recorded using a seven-point Likert-type scale (higher scores indicated stronger agreement with the statements), while demographic questions were included at the end of the survey.

### 2.3. Data Collection Procedure

A field survey was conducted among residents of the urban area of Athens. Although the study initially aimed to employ random sampling, data collection was conducted in public urban areas using voluntary participation. Therefore, the sampling procedure is more accurately described as convenience sampling. This limitation is acknowledged and considered when interpreting the findings. Participants completed the questionnaire electronically using a tablet and each participant completed the questionnaire only once. The study was approved by the Ethics Committee of Democritus University of Thrace (protocol code 31132-202/26-1-2021). All participants provided informed consent prior to participation, and anonymity and confidentiality were ensured throughout the research process.

### 2.4. Statistical Analysis

Statistical analyses were conducted using the Statistical Package for the Social Sciences (SPSS) version 29. Each statistical method was applied for a specific purpose: exploratory factor analysis was used to examine construct validity, reliability analysis assessed internal consistency, ANOVA and MANOVA tested group differences, and Pearson correlation analysis examined relationships between variables. Factor analysis was performed on both scales using exploratory factor analysis with the principal components method. To examine the validity of the PERMA questionnaire, exploratory factor analysis was conducted using principal component analysis with varimax rotation. The number of factors was determined based on eigenvalues greater than 1.00. Reliability was subsequently examined using internal consistency analysis (Cronbach’s α). In addition, descriptive statistics, analysis of variance (one-way ANOVA and MANOVA), and correlation analysis were performed. The level of statistical significance was set at *p* < 0.05. Multivariate analysis of variance (MANOVA) was conducted to examine the effects of demographic variables on multiple dependent variables simultaneously, including well-being and negotiation strategy dimensions. Pearson correlation analysis was used to examine the relationships between variables. Post hoc comparisons were conducted using the Sidak test to identify specific group differences. Only statistically significant comparisons are reported.

## 3. Results

### 3.1. Demographic Characteristics

The demographic characteristics of the sample are presented in Table 1. The sample consisted of 233 participants, with a higher proportion of women (71.2%) than men (28.8%). Most participants were aged between 19 and 59 years, with the largest group in the 40–59 age category. The majority had completed higher education, and most were employed in the private sector or self-employed. Income levels were relatively evenly distributed across low and middle categories, while most participants were either married or single (Table 1).

### 3.2. Structural Validity and Reliability of the Questionnaires

#### 3.2.1. Factor Analysis of the PERMA Well-Being Questionnaire

From the initial 29 questionnaire items, items 6 and 10 were removed because they created issues with the structural validity of the instrument. Similar observations regarding these items have been reported in the original PERMA questionnaire [40]. The principal components analysis of the remaining 27 variables revealed five factors, explaining 64.97% of the total variance. These factors were identified as: Green Commitment, Engagement–Achievement, Positive Emotions–Happiness, Negative Emotions–Loneliness, and Physical Health.

##### Mean Scores and Reliability of the PERMA Questionnaire

The results of the descriptive statistical analysis indicated relatively high mean scores across four of the five dimensions of well-being. The dimension Physical Health recorded the highest mean score (M = 5.4), whereas Negative Emotions–Loneliness presented the lowest mean score (M = 3.6). The remaining dimensions ranged between 5.0 and 5.3 in their mean values. Regarding the reliability of the PERMA questionnaire in the present study, the Cronbach’s alpha coefficient for the total of the 27 items demonstrated high internal consistency (α = 0.888). Reliability analysis for each dimension indicated that the factors Green Commitment, Engagement–Achievement, Positive Emotions–Happiness, and Physical Health showed high reliability levels (α = 0.852–0.956). The dimension Negative Emotions–Loneliness showed a relatively lower reliability coefficient (α = 0.681). However, given that research on well-being within the Greek population is still at an early stage, Cronbach’s alpha values between 0.50 and 0.60 may still be considered acceptable [42]. Furthermore, in the validation study of the PERMA Profiler for the Greek population, as well as in studies conducted in other cultural contexts, certain dimensions have demonstrated comparatively weaker psychometric properties [41] (Table 2).

#### 3.2.2. Factor Analysis of the Negotiation Strategies Questionnaire

To examine the validity of the Negotiation Strategies questionnaire, exploratory factor analysis was conducted using the principal components analysis method, followed by varimax rotation. The number of factors was determined using the criterion that eigenvalues should be greater than 1.00. The principal components analysis of the 33 variables revealed 11 factors, explaining 79.24% of the total variance. These factors were identified as: Knowledge, Negative Impact Understanding by Non-Exercise, Self-motivation, Enable, Socialization, Enhancement, Commitment, Create Pulse, Develop Relations for Encouragement, Time, and Financial. The dimension “Create Pulse” refers to the use of environmental or behavioral cues (e.g., placing sports equipment in visible locations) to trigger or remind individuals to engage in physical activity.

##### Mean Scores and Reliability of the Negotiation Strategies Questionnaire

The results of the descriptive statistical analysis indicated moderate to high mean values across the negotiation strategy dimensions. The highest mean score was recorded for the dimension Self-motivation (M = 6.2), whereas the lowest mean score was observed in the dimension Socialization (M = 3.6). The mean values of the remaining dimensions ranged between 3.8 and 5.2. Regarding the reliability of the questionnaire dimensions, the Cronbach’s alpha coefficient demonstrated high reliability across all dimensions. The highest reliability value was observed in the dimension Time (α = 0.942), while the lowest reliability value was recorded in the dimension Knowledge (α = 0.653). The reliability coefficients of the remaining dimensions ranged between α = 0.765 and α = 0.921 (Table 3).

### 3.3. Hypothesis Testing

#### 3.3.1. H1: Differences in Well-Being According to Demographic Characteristics

##### Investigation of the Effect of Occupational Status on the Dimensions of Well-Being

To examine the relationship between the independent variable occupational status and the well-being factors, a one-way analysis of variance (ANOVA) was conducted, followed by the Sidak post hoc multiple comparisons test to identify statistically significant differences between groups. The results indicated a statistically significant effect of occupational status on several dimensions of well-being. Specifically, a significant effect was observed for Engagement–Achievement, F_(7,229)_ = 4.440, *p* < 0.001, η^2^ = 0.123, indicating a moderate to large effect size. Similarly, significant effects were found for Negative Emotions–Loneliness, F_(7,229)_ = 2.323, *p* < 0.05, η^2^ = 0.068, and Physical Health, F_(7,229)_ = 3.619, *p* < 0.01, η^2^ = 0.102, both indicating moderate effect sizes. No significant effects were found for Recreation Attachment and Positive Emotions–Happiness, which showed small effect sizes (η^2^ = 0.045 and η^2^ = 0.030, respectively) (Table 4).

Specifically, it was found that participants belonging to the unemployed occupational category (M = 4.30) recorded significantly lower scores in the Engagement–Achievement dimension compared with the categories student (M = 5.02), private sector employee (M = 5.25), public sector employee (M = 5.34), self-employed (M = 5.54), homemaker (M = 5.76), and other (M = 6.19). Similarly, regarding the Physical Health factor, participants in the unemployed category (M = 4.01) reported significantly lower scores compared with the categories private sector employee (M = 5.39), student (M = 5.72), self-employed (M = 5.52), public sector employee (M = 5.63), and other (M = 6.17). Finally, concerning the Negative Emotions–Loneliness factor, no statistically significant differences were observed among occupational categories.

##### Investigation of the Simultaneous Interaction of Marital Status and Education on Well-Being Factors

A multivariate analysis of variance (MANOVA) was conducted to examine the simultaneous interaction of the independent variables marital status and education level on the factors of the dependent variable well-being. The results of the analysis revealed a statistically significant interaction effect on the factor Green Commitment, F_(9,230)_ = 2.267, *p* < 0.05. Specifically, participants who were married and graduates of vocational institutes (M = 5.927) demonstrated higher levels of green commitment compared with the categories married with a postgraduate degree (M = 4.467), single university graduates (M = 5.222), and single participants with secondary education (junior or senior high school) (M = 3.630) (Table 5).

Continuing the analysis of the main effects, a statistically significant effect of the independent variable education level was identified on the Physical Health factor, F_(3,230)_ = 3.198, *p* < 0.05. The Sidak multiple comparisons test revealed statistically significant differences between the categories postgraduate degree (M = 5.566), university graduates (M = 5.498), and vocational education (M = 4.410). Similarly, the independent variable marital status was found to have a statistically significant effect on several well-being factors: Green Commitment, F_(3,230)_ = 3.656, *p* < 0.05; Engagement–Achievement, F_(3,230)_ = 3.549, *p* < 0.05; Positive Emotions–Happiness, F_(3,230)_ = 6.697, *p* < 0.05; and Negative Emotions–Loneliness, F_(3,230)_ = 3.616, *p* < 0.05. Regarding the Green Commitment factor, the Sidak test revealed statistically significant differences between the categories cohabiting participants (M = 5.548), married participants (M = 5.323), and single participants (M = 4.587). For the Engagement–Achievement factor, the test indicated statistically significant differences between the categories married participants (M = 5.438) and single participants (M = 4.950). In the Positive Emotions–Happiness factor, statistically significant differences were found between cohabiting participants (M = 5.660), married participants (M = 5.563), and single participants (M = 4.80). Finally, regarding the Negative Emotions–Loneliness factor, statistically significant differences were observed between single participants (M = 3.947) and married participants (M = 3.329) (Table 6 and Table 7).

#### 3.3.2. H2: Differences in the Evaluation of Negotiation Strategies According to Demographic Characteristics of the Sample

##### Investigation of the Simultaneous Interaction of Age and Gender on Negotiation Strategy Factors

A multivariate analysis of variance (MANOVA) was conducted to examine the simultaneous interaction of the independent variables age and gender on the factors of the dependent variable negotiation strategies. The results of the analysis did not reveal any statistically significant interaction effect between the independent variables on the dependent variable. The analysis was subsequently continued using ANOVA, where a statistically significant main effect of age was identified on the factor Self-motivation, F_(2,233)_ = 3.806, *p* < 0.05. The Sidak multiple comparisons test revealed statistically significant differences between the age categories 60+ years (M = 6.083) and 40–59 years (M = 4.709) (Table 8).

##### Investigation of the Simultaneous Interaction of Education and Marital Status on Negotiation Strategy Factors

A multivariate analysis of variance (MANOVA) was conducted to examine the simultaneous interaction of the independent variables education level and marital status on the factors of the dependent variable negotiation strategies. The results of the analysis did not reveal any statistically significant interaction effect between the independent variables and the dependent variable. As the MANOVA analysis did not reveal statistically significant interaction effects, follow-up univariate ANOVA analyses were conducted to examine potential main effects of the independent variables on individual dependent variables. The independent variable marital status was found to have a statistically significant effect on the factor Knowledge, F_(3,233)_ = 2.824, *p* < 0.05. The Sidak multiple comparisons test revealed statistically significant differences between the categories cohabiting participants (M = 4.622) and single participants (M = 3.828) (Table 9).

#### 3.3.3. H3: Correlation Between the Dimensions of Negotiation Strategies and the Dimensions of Well-Being

The results of the correlation analysis (Pearson correlations) between the dimensions of well-being and negotiation strategies revealed statistically significant correlations among most dimensions. However, the dimension Negative Emotions–Loneliness showed a statistically significant correlation only with the Time dimension of the negotiation strategies (r = −0.133, *p* < 0.05). The correlation coefficient (r = −0.133) indicates a weak negative relationship between the variables, suggesting a limited association. No other statistically significant relationships were observed between Negative Emotions–Loneliness and the remaining negotiation strategy dimensions (Table 10).

## 4. Discussion

Regarding H1, which stated that there are differences in the evaluation of well-being according to demographic characteristics, the results partially support this hypothesis. Significant differences were observed across several demographic variables, particularly occupational status, marital status, and education level, in relation to dimensions such as Engagement–Achievement, Physical Health, and Green Commitment. These findings suggest that socio-demographic factors influence well-being outcomes, likely reflecting differences in lifestyle structure, social roles, and access to resources.

With respect to H2, which proposed that there are differences in the evaluation of negotiation strategies according to demographic characteristics, the hypothesis was partially supported. The results indicated a significant effect of age on self-motivation and an effect of marital status on the knowledge dimension. However, no significant interaction effects were observed between demographic variables, suggesting that negotiation strategies are relatively stable across population groups, with only specific dimensions being influenced by demographic characteristics.

Finally, H3, which stated that there is a correlation between the dimensions of negotiation strategies and well-being, was strongly supported. The correlation analysis revealed statistically significant positive relationships between most negotiation strategy dimensions and well-being factors, particularly Engagement–Achievement and Physical Health. These findings indicate that individuals who actively employ cognitive and behavioral strategies to overcome participation barriers are more likely to experience higher levels of well-being. The limited association observed with Negative Emotions–Loneliness suggests that negotiation strategies may be more effective in enhancing positive aspects of well-being rather than directly reducing negative emotional states.

The aim of the present study was to investigate the negotiation strategies used by residents of the urban area of Athens who participate in recreational sport activities, focusing on well-being and the potential development of outdoor green recreational spaces. In addition, the dimension “Green Commitment” was examined as a factor of well-being, and its relationship with demographic characteristics was explored in order to highlight the potential for creating sustainable outdoor green exercise environments that encourage participation in recreational sport activities.

The results demonstrated satisfactory validity and reliability for both questionnaire scales, while moderate to high mean scores were recorded across most dimensions. More specifically, within the well-being scale, the dimension Physical Health received the highest mean score, indicating the significant influence of physical and general health conditions—such as regular physical activity, proper nutrition, the elimination of unhealthy behaviors, and the maintenance of positive emotional health—on individuals’ well-being [25]. These findings are consistent with previous research highlighting the central role of physical activity in improving health outcomes and enhancing overall well-being [7,8]. Similarly, the dimensions Engagement–Achievement and Positive Emotions–Happiness were highly rated, revealing that individuals experience increased well-being when they remain focused on their exercise goals and experience feelings of satisfaction, happiness, and enjoyment through participation in recreational sport activities. These findings are aligned with the principles of the PERMA model of well-being, which emphasizes the importance of engagement, achievement, and positive emotions as fundamental components of flourishing [30,43].

The newly introduced dimension Green Commitment was also highly evaluated, highlighting the importance of the psychological, physical, and social benefits experienced by urban residents when exercising in urban green spaces. This finding supports previous studies demonstrating that exposure to natural environments promotes relaxation, stress reduction, and improved psychological well-being [22,34]. The presence of vegetation and natural elements in urban environments has been widely associated with reduced stress levels and improved emotional balance among urban populations [36,38]. Therefore, urban green spaces appear to function not only as recreational areas but also as important environmental resources that promote physical activity and well-being.

In contrast, the dimension Negative Emotions–Loneliness received relatively low scores, indicating that the absence of negative emotional states such as stress, anger, sadness, and loneliness contributes positively to individuals’ subjective well-being. This result is consistent with earlier conceptualizations of subjective well-being, which emphasize the balance between positive and negative emotional experiences [26]. Previous studies have similarly reported that participation in recreational physical activities can reduce feelings of loneliness and psychological distress while promoting emotional resilience and life satisfaction [10].

Regarding the negotiation strategies scale, most dimensions were evaluated at moderate to high levels. The dimensions Self-motivation and Enable received the highest scores, indicating that individuals’ internal motivation plays a dominant role in maintaining participation in physical activity. These findings are consistent with previous research emphasizing the importance of intrinsic motivation in sustaining exercise behavior and overcoming participation barriers [15,44]. Individuals appear to re-evaluate their personal values and actively use exercise as a behavioral strategy to overcome daily inactivity and maintain a healthy lifestyle.

The dimensions Commitment, Negative Impact Understanding by Non-Exercise, and Time were evaluated at moderate levels, suggesting that individuals may still face difficulties in fully integrating exercise into their daily routines. Similar findings have been reported in previous research indicating that lack of time, competing priorities, and limited awareness of the consequences of physical inactivity often function as barriers to regular physical activity participation [14,20].

Lower scores were recorded for the dimensions Enhancement, Knowledge, and Develop Relations for Encouragement, suggesting that individuals do not frequently apply strategies related to self-reinforcement or social support in order to maintain their participation in physical activity. Previous studies have highlighted that social encouragement and access to information can significantly influence individuals’ motivation to engage in recreational sport activities [16,45]. The relatively lower evaluation of these dimensions in the present study may indicate that urban residents rely more heavily on internal motivational processes than external sources of encouragement.

Finally, the dimensions Create Pulse, Financial, and Socialization received the lowest evaluations, indicating limited use of external environmental cues and social strategies to support exercise participation. For example, individuals may not adopt behavioral strategies such as placing sports equipment in visible locations or planning exercise sessions with friends to increase motivation. This example refers to behavioral cueing strategies, where environmental prompts (e.g., placing sports equipment in visible locations) serve as reminders that facilitate engagement in physical activity. These findings are consistent with previous research suggesting that financial limitations and lack of social support can function as barriers to participation in recreational physical activities [17].

These findings are supported by recent studies emphasizing the importance of urban green environments in shaping recreational experiences and well-being outcomes. For example, ref. [46] highlighted that environmental quality and user perception significantly influence recreational experiences in urban river corridors, particularly through social and aesthetic factors. Similarly, ref. [47] demonstrated a growing demand for year-round engagement in urban green exercise, suggesting that accessibility and environmental conditions play a key role in sustained participation. From a human–nature interaction perspective, ref. [48] found that engagement with urban ecological spaces positively affects well-being, reinforcing the importance of environmental context in recreational behavior. In addition, ref. [49] showed that urban green areas contribute not only to physical health but also to environmental awareness and broader public health outcomes. Finally, ref. [50] emphasized the role of public perception in shaping the use and value of urban recreational spaces, indicating that individuals’ subjective experiences influence their engagement with such environments. Taken together, these findings support the present study’s results, suggesting that the interaction between behavioral strategies and environmental context plays a crucial role in promoting well-being in urban settings.

### Limitations and Future Research

The sample of the present study consisted of 233 participants, a number that cannot fully represent the entire population of residents living in the urban area of Athens. Additionally, the sample selection was based on random participation. Future studies should aim to include larger and more representative samples from different districts of the Municipality of Athens and surrounding areas, in order to examine potential differences in levels of well-being, green commitment, and negotiation strategies among residents. The present study also provided the opportunity to examine the dimension “Green Commitment” as a factor of well-being. However, further research is recommended in order to investigate the structural validity and reliability of this new questionnaire dimension in different populations and contexts. Although the study examined several demographic variables, socioeconomic status was not directly measured. Given its potential influence on access to green spaces and opportunities for physical activity, future research should incorporate this variable to provide a more comprehensive understanding of participation behaviors.

Moreover, this study represents one of the first attempts to combine the Negotiation Strategies Scale for participation in recreational sport activities with the PERMA Profiler well-being questionnaire. Future research could further examine the causal relationships between negotiation strategy factors and well-being dimensions, particularly in relation to Green Commitment. It is also recommended that future research employs mixed research designs, combining quantitative and qualitative data in order to provide a more holistic understanding of green recreation and its relationship with well-being. Additionally, longitudinal studies could be conducted to evaluate the long-term impact of green recreation on the lives of urban residents.

The present study has several strengths. It integrates behavioral, environmental, and psychological perspectives within a single framework, examines a real urban population, and introduces the concept of Green Commitment as a novel dimension of well-being in the context of urban green environments. Finally, future research should aim to develop and empirically validate a comprehensive theoretical model of urban green recreation, examining its benefits at both the social level and the level of sustainable personal development. Such a model could serve as an important educational tool for promoting well-being and quality of life across all age groups, contributing to the cultivation of a healthier and more sustainable urban society.

## 5. Conclusions

In conclusion, the findings demonstrate that negotiation strategies play a key role in supporting participation in recreational sport and enhancing well-being among urban residents. Engagement with urban green environments further contributes to these outcomes, highlighting the importance of accessible and well-designed green spaces in promoting healthy and active lifestyles. The present study examined the roles of negotiation strategies in recreational sport participation and their relationships with well-being among residents of the urban area of Athens. The findings indicate that demographic characteristics such as occupation, education level, age, and marital status influence both the evaluation of well-being and the use of negotiation strategies related to participation in physical activity.

The results also highlight the importance of urban green recreational environments, as participants who engaged in exercise in green spaces reported higher levels of well-being, particularly in terms of physical health, positive emotions, and engagement in recreational activities. In addition, the newly examined dimension Green Commitment appears to be an important component of well-being in urban populations, emphasizing the value of accessible green spaces for promoting healthy lifestyles.

The findings highlight the importance of both recreational sport participation and general physical activity in promoting well-being among urban populations. Urban policies and planning strategies should prioritize access to green spaces and recreational infrastructure, enabling individuals to overcome barriers and engage in both structured and unstructured forms of physical activity.

### Practical Implications

Within the context of developing social policies aimed at improving urban quality of life, the development and enhancement of urban green recreational parks emerge as a key priority. The systematic maintenance and utilization of neglected parks in Athens, such as the Academy of Plato Park, as well as the creation of new green recreational areas accessible to multiple neighborhoods of the city, are strongly recommended. Urban green spaces, given their well-documented benefits for well-being and physical and psychological health, proved particularly important during the recent challenging period of the COVID-19 pandemic, as they provided opportunities for emotional release, social interaction, and the strengthening of positive emotions. Equally important are public and private initiatives aimed at providing recreational sport programs in outdoor green environments so that individuals of all age groups can benefit from contact with nature. Finally, strategic actions are recommended to raise public awareness of the benefits of recreational physical activity in green environments, with the ultimate goal of cultivating a culture that promotes sustainable living in modern metropolitan cities such as Athens.

## Figures and Tables

**Table 1:** Demographic characteristics of the sample (N = 233).

Variable	Category	N	%
Gender	Male	67	28.8
	Female	166	71.2
Age Group	19–39 years	109	46.8
	40–59 years	115	49.4
	60+ years	9	3.9
Education Level	Secondary education	28	12
	Vocational training	31	13.3
	University	91	39.1
	Postgraduate/PhD	83	35.6
Employment Status	Private sector	88	37.8
	Self-employed	70	30
	Public sector	32	13.7
	Unemployed	10	4.3
	Retired	7	3
	Household	6	2.6
	Other	20	8.6
Income	<€20,000	111	47.6
	€20,000–€60,000	102	43.8
	>€60,000	20	8.6
Marital Status	Married	90	38.6
	Single	86	36.9
	Cohabiting	39	16.7
	Divorced	18	7.7

**Table 2:** Mean scores and Cronbach’s α coefficients of the PERMA well-being questionnaire.

PERMA Well-Being Factors	Mean	SD	Alpha	Items
Green Commitment	5.0	1.5	0.956	6
Engagement–Achievement	5.3	0.92	0.885	9
Positive Emotions–Happiness	5.3	1.1	0.852	5
Negative Emotions–Loneliness	3.6	1.1	0.681	4
Physical Health	5.4	1.1	0.867	3

**Table 3:** Mean scores and Cronbach’s α coefficients of the Negotiation Strategies questionnaire.

Negotiation Strategies Factors	Mean	SD	Alpha	Items
Knowledge	4.1	1.2	0.653	3
Negative Impact Understanding by Non-Exercise	4.8	1.6	0.891	3
Self-motivation	6.2	1.0	0.830	3
Enable	5.2	1.5	0.848	3
Socialization	3.6	1.5	0.765	3
Enhancement	4.4	1.4	0.779	3
Commitment	4.9	1.5	0.844	3
Create Pulse	3.9	1.6	0.835	3
Develop Relations for Encouragement	4.0	1.5	0.810	3
Time	4.6	1.6	0.942	3
Financial	3.8	1.8	0.921	3

**Table 4:** Analysis of variance between occupational categories and well-being factors.

PERMA Factors		Student	Private Sector Employee	Public Sector Employee	Self-Employed	Unemployed	Household	Retired	Other	F	Post Hoc
Engagement–Achievement	Mean	4.91	5.25	5.34	5.54	4.30	5.76	5.02	6.19	F = 4.440 *p* < 0.05	1–8 *, 2–5 *, 3–5 *, 4–5 *, 5–6 * 5–8 *
Physical Health	Mean	5.72	5.39	5.63	5.52	4.01	5.11	4.50	6.17	F = 3.619 *p* < 0.05	1–5 *, 2–5 *, 3–5 *, 4–5 *, 5–8 *

* Significant differences were observed between categories.

**Table 5:** Multivariate analysis of variance between marital status and education categories and well-being factors.

PERMA Factor		Married—College Graduate	Married—Postgraduate Degree	Single—University Graduate	Single—Secondary Education	F
Green Commitment	Mean	5.382	4.467	3.630	5.222	F = 2.297 *p* < 0.05

**Table 6:** Analysis of variance between education categories and well-being factors.

PERMA Factor		Secondary Education	Vocational Education	University Graduate	Master’s/PhD Degree Holders	F	Post Hoc
Physical Health	Mean	5.438	5.581	4.985	4.905	F = 3.198 *p* < 0.05	2–3 *, 2–4 *

* Means where the difference is located.

**Table 7:** Analysis of variance of well-being factors across marital status categories.

PERMA Factors		Married	Divorced	Single	Cohabiting	F	Post Hoc
Green Commitment	Mean	5.323	5.451	4.587	5.548	F = 3.656 *p* < 0.05	1–3 *, 3–4 *
Engagement–Achievement	Mean	5.438	5.358	4.950	5.460	F = 3.549 *p* < 0.05	1–3 *
Positive Emotions–Happiness	Mean	5.563	5.092	4.80	5.66	F = 6.697 *p* < 0.05	1–3 *, 3–4 *
Negative Emotions–Loneliness	Mean	5.325	4.873	5.315	5.504	F = 3.616 *p* < 0.05	1–3 *

* The differences revealed between these categories.

**Table 8:** Analysis of variance between age categories and negotiation strategy factors.

Negotiation Strategy Factor	Age	18–39	40–59	60+	F	Post Hoc
Commitment	Mean	5.113	4.709	6.083	F = 3.806 *p* < 0.05	2–3 *

* Means where the difference is located.

**Table 9:** Analysis of variance between marital status categories and negotiation strategy factors.

Negotiation Strategy Factor		Married	Divorced	Single	Cohabiting	F	Post Hoc
Knowledge	Mean	4.118	3.815	3.828	4.622	F = 2.824 *p* < 0.05	3–4 *

* Means where the difference is located.

**Table 10:** Correlation results between the dimensions of negotiation strategies and the dimensions of well-being.

	PERMA Factors				
Negotiation Strategy Factors	Green Commitment	Engagement–Achievement	Positive Emotions–Happiness	Negative Emotions–Loneliness	Physical Health
Knowledge	0.297 **	0.419 **	0.254 **	ns	0.199 **
Negative Impact Understanding by Non-Exercise	0.245 **	0.324 **	0.157 *	ns	0.242 **
Self-motivation	0.271 **	0.353 **	0.236 **	ns	0.320 **
Enable	0.319 **	0.413 **	0.229 **	ns	0.410 **
Socialization	0.311 **	0.275 **	0.197 **	ns	0.243 **
Enhancement	0.304 **	0.346 **	0.274 **	ns	0.368 **
Commitment	0.336 **	0.399 **	0.293 **	ns	0.391 **
Create Pulse	0.133 *	0.283 **	0.203 **	ns	0.296 **
Develop Relations for Encouragement	0.266 **	0.194 **	0.205 **	ns	ns
Time	0.182 **	0.338 **	0.259 **	−0.133 *	0.469 **
Financial	0.239 **	0.283 **	0.186 **	ns	0.341 **

** *p* < 0.001, * *p* < 0.05, ns: not significant.

## Data Availability

The data presented in this study are available on request from the corresponding author due to privacy and ethical restrictions.
